# Elevation-dependent responses of tree mast seeding to climate change over 45 years

**DOI:** 10.1002/ece3.1210

**Published:** 2014-08-28

**Authors:** Robert B Allen, Jennifer M Hurst, Jeanne Portier, Sarah J Richardson

**Affiliations:** 1 Landcare ResearchPO Box 40, Lincoln, 7640, New Zealand; 2 Centre d’Etude de la Forêt, Université du Québec à MontréalC.P. 8888, Montréal, Québec, H3C 3P8, Canada

**Keywords:** Beech, environmental gradient, intraspecific, long-term data, New Zealand, *Nothofagus solandri* var. *cliffortioides*, seed production, resources, time series

## Abstract

We use seed count data from a New Zealand mono-specific mountain beech forest to test for decadal trends in seed production along an elevation gradient in relation to changes in climate. Seedfall was collected (1965 to 2009) from seed trays located on transect lines at fixed elevations along an elevation gradient (1020 to 1370 m). We counted the number of seeds in the catch of each tray, for each year, and determined the number of viable seeds. Climate variables were obtained from a nearby (<2 km) climate station (914-m elevation). Variables were the sum or mean of daily measurements, using periods within each year known to correlate with subsequent interannual variation in seed production. To determine trends in mean seed production, at each elevation, and climate variables, we used generalized least squares (GLS) regression. We demonstrate a trend of increasing total and viable seed production, particularly at higher elevations, which emerged from marked interannual variation. Significant changes in four seasonal climate variables had GLS regression coefficients consistent with predictions of increased seed production. These variables subsumed the effect of year in GLS regressions with a greater influence on seed production with increasing elevation. Regression models enforce a view that the sequence of climate variables was additive in their influence on seed production throughout a reproductive cycle spanning more than 2 years and including three summers. Models with the most support always included summer precipitation as the earliest variable in the sequence followed by summer maximum daily temperatures. We interpret this as reflecting precipitation driven increases in soil nutrient availability enhancing seed production at higher elevations rather than the direct effects of climate, stand development or rising atmospheric CO_2_ partial pressures. Greater sensitivity of tree seeding at higher elevations to changes in climate reveals how ecosystem responses to climate change will be spatially variable.

## Introduction

Predicting the consequence of a changing climate on the distribution, structure, composition, and function of forest ecosystems is necessary to ensure forests can be managed to provide services essential to society (Millar et al. 2007). One means of determining the consequence of a changing climate is by analyzing spatial and temporal variability in tree demographic processes and their relationships to climate (e.g., Clark et al. 2011). An increased frequency of drought, for example, has been linked to increased adult-tree mortality, which may influence the persistence of a species at a location (e.g., Van Mantgem and Stephenson 2007; Allen et al. 2010). The distributional response of a tree species to a changing climate may also be driven by regeneration processes as these control the ability of tree species to spread to new locations (Clark et al. 2001; Ibáñez et al. 2008). The persistence or spread of tree species may also be influenced by temporal trends in seed production, and the supply of propagules, in response to a change in climate. Because seed production not only controls tree population demography but also, for example, consumer dynamics (e.g., Janzen 1971), understanding drivers of seed production is critical to determining forest ecosystem responses to climate change.

Distinctive interannual patterns of tree seed production include spatial synchronicity (e.g., Burrows and Allen 1991; Koenig and Knops 2000; Fearer et al. 2008), periodicity (e.g., Caron and Powell 1989; Sork et al. 1993; Allen et al. 2012; but see Greene and Johnson 2004), high variability (e.g., Silvertown 1980; Herrera et al. 1998; Greene and Johnson 2004), and negative temporal autocorrelation (e.g., Koenig et al. 2003; Crone et al. 2011). Several hypotheses have been proposed to explain the development of synchronous and highly variable seed production (Kelly 1994). Predator satiation (e.g., Janzen 1971) and increased pollination efficiency (e.g., Nilsson and Wästljung 1987) are considered the best-supported evolutionary explanations, while climate and resource dynamics act as important proximate factors (Koenig and Knops 2005; Crone et al. 2009; Smaill et al. 2011; Sala et al. 2012; Tanentzap et al. 2012; Danielson and Frommer 2013). While interannual patterns in tree seed production have been a focus of much research, few studies have determined decadal trends (but see Kullman 2002; Richardson et al. 2005).

Interannual variation in seasonal climate often relates to interannual variation in tree flowering (e.g., Law et al. 2000; Cook et al. 2012) and seed production (e.g., Eis 1973; Wardle 1984). A wide range of climate variables have been matched to these phases. Cool temperatures and increased moisture availability during what appears to be a resource priming accumulation of reserves, approximately 2 years prior to seedfall, have commonly been shown to increase seed production across populations of various tree species (e.g., Lowry 1966; Van Vredenburch and la Bastide 1969; Eis 1973; Piovesan and Adams 2001, 2005; Richardson et al. 2005). Relatively high temperatures during floral primordia development, approximately 1 year prior to seedfall (e.g., Van Vredenburch and la Bastide 1969; Eis 1973; Schauber et al. 2002; Övergaard et al. 2007), and relatively high temperatures postflowering, immediately before seeding (e.g., Richardson et al. 2005; Smaill et al. 2011), can also increase seed production. Combinations of these precipitation and temperature variables sometimes explain most of the interannual variation in tree seed production. In New Zealand mountain beech (*Nothofagus solandri* var. *cliffortioides* (Hook.f.) Poole) forests, for example, coefficients of determination ranged between 0.84 and 0.92 for models explaining total seed mass production from such variables (Smaill et al. 2011). The sensitivity of seed production to seasonal climate can be modulated by soil resource availability (Smaill et al. 2011; Tanentzap et al. 2012). A question that remains is whether there have been decadal changes in climate variables and what the consequences are of any change for seed production. We hypothesize that seed production responses to any climate change will not be spatially uniform and will vary along gradients in resource availability.

Overall, New Zealand has experienced only modest changes in climate over recent decades compared with other parts of the world. Annual mean, maximum, and minimum daily temperatures have increased by 0.2, 0.1, and 0.4°C, respectively (1951–1998), but changes are regionally variable because mountain ranges influence climatic patterns (Salinger and Mullan 1999; Salinger and Griffiths 2001). Changes in precipitation (1951–1998) also vary regionally, although increases in precipitation have been recorded over much of the South Island because of an increase in west to southwest winds (Manton et al. 2001; Salinger and Griffiths 2001). While these decadal changes in climate have been small relative to interannual variability (Salinger and Griffiths 2001), we might expect such changes to affect seed production trends where they have strong climatic relationships. Increases in precipitation might more strongly influence total seed production (somewhat equivalent to number of flowers) because of its relationship to resource priming. Increases in temperature, particularly postflowering, might more strongly influence viable seed production as viable seed production reflects, in part, the influence of postpollination factors during a reproductive period (e.g., Allen and Platt 1990).

We use 45 years of seed count data in a population-level (sensu Kelly 1994) test of decadal trends in total and viable seed production along an elevation gradient in a mountain beech forest in relation to changes in climate. Recently, Kelly et al. (2013) concluded that mast seeding will be unaffected by gradual increases in temperature, whereas Pearse et al. (2014) opposed this view and suggested such climate changes will be proven to influence mast seeding when long-term quantitative data and appropriate analyses are forthcoming. Mountain beech forms mono-specific forests that dominate extensive areas in the drier montane and subalpine forests in eastern parts of New Zealand between 36 and 46°S (Wiser et al. 2011). Because interannual variability in seed production and climate is large, long-term data are required to partition out what are likely to be subtle decadal trends. We test the following: (1) for a decadal trend in total and viable seed production, and if this is more pronounced with increasing elevation, and (2) whether changes in precipitation and temperature are occurring in the mountain beech forest at resource priming, primordial development, or postflowering times. A more favorable climate for seed production in a stressed environment (higher elevations) may reduce tree recovery times from seeding and increase average seed production (Kelly and Sork 2002). Finally, we test the following: (3) whether any decadal trends in total seed production are better explained by changes in resource priming precipitation and temperature or whether viable seed production is better explained by changes in primordia development or postflowering temperature. We also hypothesize that the influence of individual climate variables on total and viable seed production will be additive during the phases of reproduction.

## Materials and Methods

### Study area and species

Stands were selected within the extensive mountain beech forests of the Craigieburn Range (43°13′S, 171°69′E), South Island, New Zealand. Mountain beech is the only tree species forming the natural forest of the Craigieburn Range from 800-m elevation up to tree line at c. 1370-m elevation. Climate observations were available from two climate stations (1964 to 1979) located within 2 km of the Craigieburn Range stands: Craigieburn Forest (914 m elevation) and Ski Basin (1550 m elevation). Mean annual temperature at Craigieburn Forest was 8.0°C, with the highest mean monthly temperature occurring in February (13.9°C) and the lowest in July (2.0°C; McCracken 1980). The lapse rate of mean annual temperature between Craigieburn Forest and Ski Basin was 0.66°C per 100 m of elevation. Mean annual precipitation at Craigieburn Forest was 1447 mm, with February and March receiving <100 mm (McCracken 1980). Mean annual precipitation at Ski Basin was 139 mm higher than at Craigieburn Forest. Soils in the study area are acidic and low in nitrogen and cation availability (Allen et al. 1997; Clinton et al. 2002), the availability of which declines with increasing elevation (Coomes and Allen 2007).

Mountain beech is a long-lived (250–350 years) evergreen tree species. The species is monoecious, with wind-pollinated flowers, and produces a single-seeded nut enclosed in a cupule. Reproduction spans two growing seasons. In the first season, floral primordia are laid down in dormant buds soon after they begin to form. Flowering occurs in the second season, and the timing is strongly influenced by site conditions, so that flowering can occur in late October at 450-m elevation and in early January at tree line (Wardle 1984). The nuts ripen and seeds are shed c. 6 months after pollination. Mountain beech annual total seed production (at a site) ranges from <10 (15% of years) to >6000 (15% of years) seeds m^−2^ (Allen and Platt 1990; Richardson et al. 2005; Allen et al. 2012). While annual total seed production does not vary with elevation, annual viable seed production does decline to a limited degree (Wardle 1984; Allen et al. 2012). Even at higher elevations, there is little evidence for a marked bimodality in the frequency distribution of seed crop size (Allen et al. 2012).

Mountain beech stands also exhibit a decline in biomass, net productivity, height, and mortality with increasing elevation, but a small increase in stem density and basal area (e.g., Benecke and Nordmeyer 1982; Harcombe et al. 1998; Richardson et al. 2005; Coomes and Allen 2007). Benecke and Nordmeyer (1982) showed that net annual primary production declines from 33.6 *t*·ha^−1^·year^−1^ at 1000-m elevation to 18.0·*t*·ha^−1^·year^−1^ at 1320-m elevation in the Craigieburn Range. This is likely because growing season temperatures increasingly restrict growth with elevation due to the influence of cool air temperatures. However, competition for soil nutrients (predominantly nitrogen) also limits mountain beech growth and seed production, with competition for nutrients appearing most intense near tree line (Davis et al. 2004; Platt et al. 2004; Coomes and Allen 2007).

### Data collection

Mountain beech seedfall was collected along transect lines within stands at 1050-, 1190-, and 1340-m elevation (each approximately 0.3 km apart) representing a strong productivity gradient in the Craigieburn Range. Each line included eight seed trays arranged c. 40 m apart. Seed trays were funnel-shaped with a catch area of 0.28 m^2^. Seedfall at these three elevations was measured from 1965 to 2009 (see details in Allen and Platt 1990). In 1973, a further six lines of seed trays were added at 1020-, 1095-, 1145-, 1240-, 1295-, and 1370-m elevation, each line of which contained only two seed trays. These seed trays were measured from 1973 to 2009. Canisters beneath trays were emptied at intervals between March and September, the period of seedfall. We counted the number of nuts in the seed catch of each tray, for each year. The number of viable nuts (intact endosperm) was determined in most years by cutting each nut with a scalpel to examine the endosperm. However, in years with high seed production, viability was instead determined by floating the nuts in 99% ethanol (tested against cutting by Ledgard and Cath (1983). We used data from the three lines spanning 45 years (1965 to 2009) to examine temporal trends related to climate and the nine lines spanning 37 years (1973 to 2009) to further examine the influence of elevation on total and viable seed production.

From 1964 to 2009, climate data were only collected at the Craigieburn Forest climate station. Daily precipitation was initially measured from a weighing-bucket rainfall gauge and weekly charts and then using a tipping rain gauge connected to a CR10 data logger (Campbell Scientific, Logan, UT). Daily maximum and minimum temperatures were initially measured from thermometers and then from calibrated thermocouples connected to the data logger (both within a 1.5-m Stevenson screen). Mean daily temperature was calculated as the average of the daily maximum and minimum temperatures. Missing daily values (< 1%) were calculated using the average of the previous and following days, where there were ≤5 missing values in a row, or the average of the previous and next year for the same day where >5 consecutive daily values were missing.

### Data analyses

**Figure 1:**
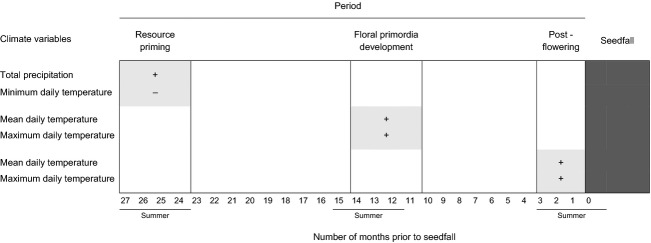
Climate variables are given for three periods during the reproductive cycle. Periods given are those which strongly correlate with interannual variation in total and viable mountain beech seed production. Positive (+) and negative (−) correlations are indicated.

For total and viable seed production, at each of three elevations, we determined the mean annual seed production (per square meter) of the eight trays in each year from 1965 to 2009. To accommodate the non-normal distribution and zero values, seed production data were transformed using log_10_ (seedfall + 1) for all analyses (e.g., Richardson et al. 2005; Fearer et al. 2008; Koenig and Knops 2014). Six climate variables were calculated as the total or mean of the daily measurements (1964 to 2009) at Craigieburn Forest, using periods within each year known to strongly correlate with interannual variation in mountain beech seed production (Fig. 1): resource priming using total precipitation and mean daily minimum temperature from December to March 2 years prior to seedfall (Richardson et al. 2005; Smaill et al. 2011); floral primordia development using mean daily temperature and mean daily maximum temperature from January to April 1 year prior to seedfall (Allen and Platt 1990; Richardson et al. 2005; Smaill et al. 2011); and postflowering using mean daily temperature and mean daily maximum temperature from December to February immediately before seed production (Allen and Platt 1990; Richardson et al. 2005). Resource priming and floral primordia temperature effects on mountain beech total and viable seed production are relatively uniform with elevation whereas resource priming precipitation and postflowering temperature effects are more pronounced at higher elevations (Allen and Platt 1990; Richardson et al. 2005).

Our first analysis determined whether there were temporal trends in seed production over the longer period (1965 to 2009) or changes in climate variables (1964 to 2009). We used generalized least squares (GLS) as implemented in the *nlme* package in R (Crawley 2003; Pinheiro et al. 2013). For seed production (both mean annual total and viable), we fitted separate GLS models for seed production at each of the three elevations. When examining whether there was a temporal trend (i.e., an effect of year) in each dependent variable (seed production and climate variables), we initially compared two alternative models, both with and without a lag-1 (first order) autoregressive correlation structure, to account for the possibility that low years follow high years (e.g., Crawley 2003; Crone et al. 2011). We determined the Akaike information criterion (AIC) for each model and selected the most supported model for each dependent variable. Models with first-order autoregressive correlation structures were best supported for seed production (e.g., reduction in AIC of between −3.3 and −16.4 for models including a lag-1 autoregressive correlation structure compared to models without), so for subsequent analyses we used that model form. Models with ΔAIC < −3, compared to the null model, are generally considered strongly supported (Burnham and Anderson 2002). For climate variables, models without autoregressive correlation structures were best supported (e.g., ΔAIC of between −1.2 and −2.0 for models including a lag-1 autoregressive correlation structure compared to models without). We also fitted a set of alternate GLS models to determine whether there was an effect of elevation, or an interaction between year and elevation, on total and viable seed production. For each model, we determined the reduction in AIC (ΔAIC) from that of a null model (a model without any explanatory variables).

We then applied a similar regression approach, using GLS, to the seed production data from all nine elevations from the shorter period (1973 to 2009), to better determine whether any temporal trends in seeding varied with elevation. We standardized the number of seed trays on each transect line by only including two randomly selected trays from the lines measured from 1965 to 2009 at 1050-, 1190-, and 1340-m elevation (for adequacy of two trays, see Burrows and Allen 1991). For all nine elevations, we determined the mean annual total and viable seed production (per square meter). We again fitted a regression separately for each elevation, to examine whether there was an effect of year on the mean number of total and viable seeds produced at each elevation. We determined the slope and significance (*P*-value) of the time trend for total and viable seed production at each elevation and examined the regression coefficients to assess how the rate of change (i.e., the slope) varied with elevation.

A final set of GLS regressions were used to determine whether any temporal trends in total or viable seed production were explained by any of the climate variables that displayed significant temporal trends in our earlier analyses. These analyses were run for mean annual seed production at the three elevations over the 1966–2009 period (c.f. 1965 to 2009 in earlier analyses) because although climate data collection began in 1964, calculation of the climate variables during resource priming required data 2 years prior to seed production. Values for a data gap in December 1963 (needed to calculate climate variables for the resource priming period) were generated by taking a mean of December records for the 1964–2009 period. To examine whether there was a temporal trend in seeding over and above that explained by the climate variable, we used each of the climate variables as fixed effects in a regression to predict seed production, and compared the AIC to further regressions that included each climate variable in addition to year. To determine whether a climatic influence on seed production varied with elevation, we also fitted models with interactions between climate variables and elevation. We compared each of the 12 models with a null model using ΔAIC. We determined *P*-values for each variable included in each regression. Regression coefficients (± standard error) are given in Table S1. Lastly, to test whether the influence of climate variables on seed production was additive during the sequence of reproductive phases, we performed a set of GLS regressions with all possible combinations of variables and determined the ΔAICs and *P*-values.

## Results

**Figure 2:**
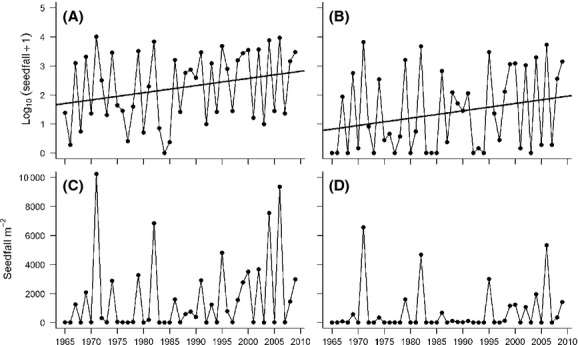
Mean annual total and viable seed production (seedfall m^−2^) at 1340-m elevation over a 45-year period (1965–2009). (A) total seed production (transformed using log_10_(seedfall +1)) showing linear regression relationship between log-transformed data and time, fitted using generalized least squares regression (GLS); (B) viable seed production (transformed using log_10_(seedfall +1)) showing linear regression relationship between log-transformed data and time, fitted using GLS. (C) total seed production (raw data); (D) viable seed production (raw data).

Mean total and viable mountain beech seed production increased significantly between 1965 and 2009 only at the highest of the three elevations, that is, 1340-m elevation (Table 1; Fig. 2A and B). Reductions in Akaike information criterion (ΔAIC) for models including “year” were –3.93 and –3.72 for total and viable seed production, respectively. The regression coefficients for total and viable seed production at 1340-m elevation were both 0.025 (Table 1), indicating both increased over time. Increased total seed production at 1340-m elevation was largely driven by the increasing frequency of moderate-to-high seedfall years (>1000 seeds m^2^; Fig. 2A and C). Increased viable seed production at 1340-m elevation was driven by more years with moderate seedfall and fewer years with zero seedfall (Fig. 2B and D). The autoregressive correlation term showed that mountain beech seed production was negatively correlated among consecutive years (Fig. 2A and B; i.e., across all models, high years were likely to be followed by low years, with the autoregressive model estimating parameter Phi ranging between –0.33 and –0.58).

**Table 1:** Temporal trends in mean annual total and viable seed production (1965 to 2009) at three elevations (1050, 1190, and 1340 m) determined using generalized least squares regression. Slope, *P*-value, and the reduction in Akaike information criterion (ΔAIC) from a null model are given

	Total seed production			Viable seed production		
Elevation (m)	Slope	ΔAIC	*P*-value	Slope	ΔAIC	*P*-value
1050	0.011	0.61	0.251	0.013	0.02	0.171
1190	0.013	0.09	0.177	0.017	−1.36	0.073
1340	0.025	−3.93	0.015	0.025	−3.72	0.018

**Figure 3:**
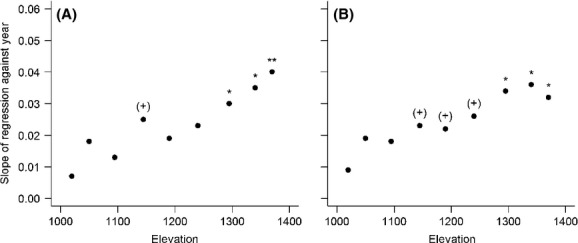
Strength of temporal trend in seed production at nine elevations (1973 to 2009). Each point represents the slope at one elevation determined from a generalized least squares regression (GLS) between year and the log-transformed seed production data (A, total seed production and B, viable seed production). For each elevation, a significant slope is denoted as (+), *P* < 0.1; *, *P* < 0.05; **, *P* < 0.01.

Between 1973 and 2009, mean total and viable seed production increased significantly on seed tray lines at the top three of nine elevations – between 1295- and 1370-m elevation (Fig. 3A and B). For viable seed production, there was also weaker increases (*P* < 0.1) between 1145- and 1240-m elevation (Fig. 3B). Regression coefficients gradually increased across the nine elevations indicating that seed production increased more at higher elevations than at lower elevations. Regression coefficients for total and viable seed production at 1340-m elevation were higher for the more recent shorter period between 1973 and 2009 (Fig. 3A and B) than the longer period between 1965 and 2009 (Table 1) suggesting a strengthening of the increasing seed production trend. When total and viable seed production from the nine elevations (between 1973 and 2009) was instead analyzed in one model with elevation as an explanatory variable, there was a significant temporal trend (ΔAIC of −23.7 and −25.7, respectively), although not year by elevation interactions (ΔAIC of 1.1 and 0.4, respectively).

**Figure 4:**
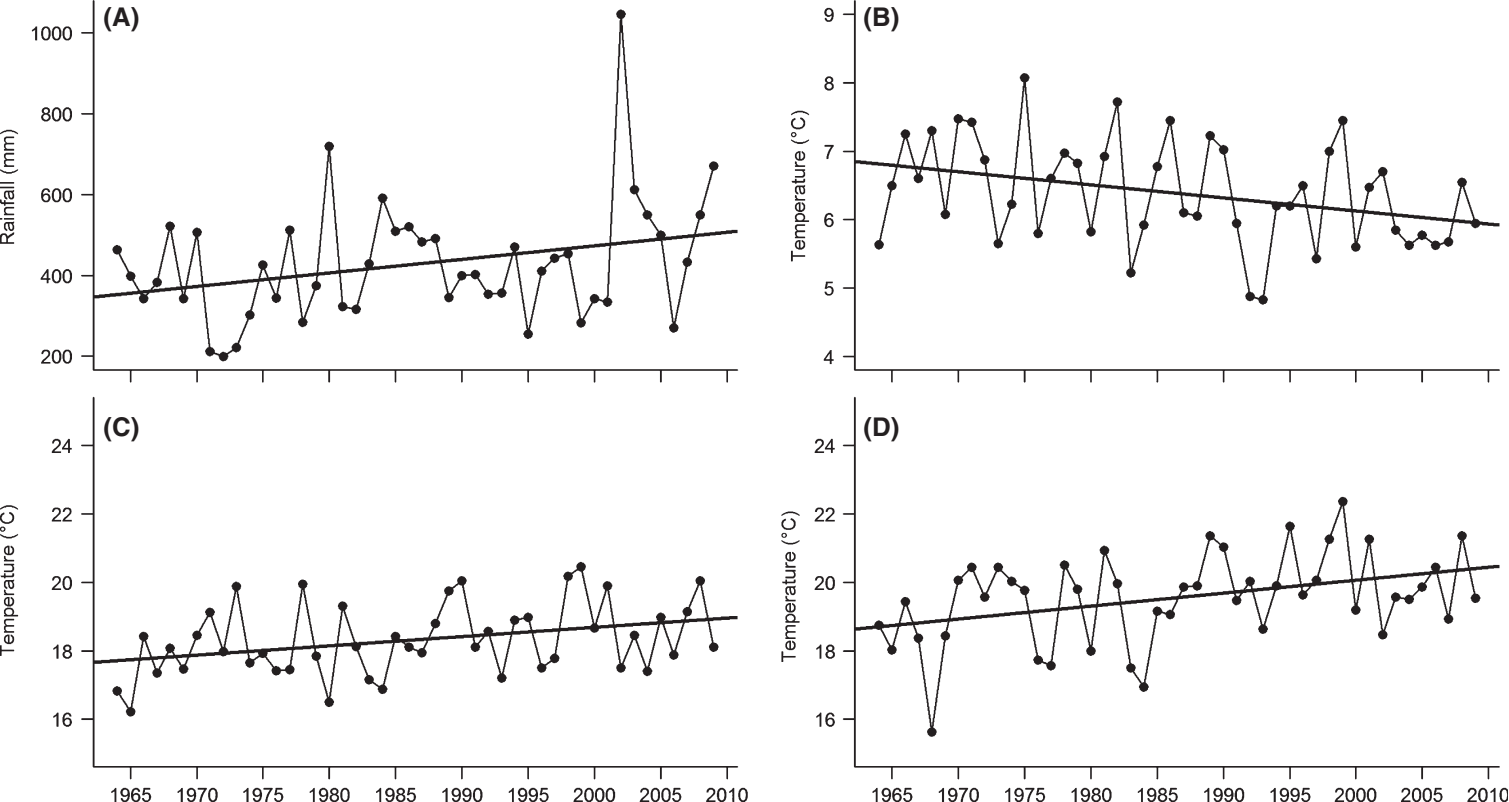
Significant temporal changes in climate variables (1964 to 2009). Climate variables were as follows: A, precipitation during resource priming; B, mean daily minimum temperature during resource priming; C, mean daily maximum temperature during primordia development; and D, mean daily maximum temperature postflowering. Significant linear relationships between time and each climate variable are illustrated, fitted using generalized least squares regression (GLS).

Resource priming total precipitation and mean daily minimum temperature increased and decreased, respectively, between 1964 and 2009 (ΔAIC of −2.3 and −3.5, respectively, compared to null models, Table 2; Fig. 4A and B). In contrast to resource priming mean daily minimum temperature, floral primordia development mean daily maximum temperature increased. Postflowering mean daily maximum temperature showed the strongest temporal trend of any temperature variable (ΔAIC of −5.5, Fig. 4D; Table 2). It is of interest to note that the increasing mean daily maximum temperatures over summers are balanced somewhat by the decreasing mean daily minimum temperatures over summers, so there was no significant change in mean daily temperatures over the summer (Table 2).

**Table 2:** Temporal changes in climate variables (1964 to 2009) determined using generalized least squares regression. Climate variables were precipitation (Prec) or temperature (as mean daily minimum (*T*_min_), mean daily (*T*_mean_), or mean daily maximum (*T*_max_) for resource priming (RP), primordia development (PD), and postflowering (PF)) periods. Regression slopes, reductions in Akaike information criterion (ΔAIC) from a null model, and *P*-values are given for each climate variable

Climate variable	Slope	ΔAIC	*P*-value
PrecRP	3.340	–2.3	0.043
*T*_min_RP	−0.019	−3.5	0.023
*T*_max_PD	0.027	−4.0	0.018
*T*_mean_PD	0.002	1.9	0.796
*T*_max_PF	0.038	−5.5	0.008
*T*_mean_PF	0.010	0.8	0.283

The four climate variables that displayed significant temporal changes predicted both total and viable seed production between 1966 and 2009 using the data from three elevations (Table 3). Total and viable seed production were both positively related to total precipitation at resource priming, mean daily maximum temperature at primordia development, and mean daily maximum temperature postflowering (e.g., regression coefficients in Table S1). In contrast, mean daily minimum temperature at resource priming was negatively related to total and viable seed production (Table S1). Models that included the effect of year in addition to each climate variable did not receive support (based upon AIC) when compared with the simpler models (i.e., models with each of the four climate variables alone were always within 2 AIC units of models including both a climate variable and year, Table 3). This, along with significant *P*-values for each of the four climate variables, but not for year when also included, suggested that climate variation explained the temporal trend in total and viable seed production (Table 3). For total seed production between 1966 and 2009, ΔAIC and *P*-values for the climate variable interaction with elevation suggest the influence of each climate variable increased with elevation (Table 3, Table S1). In contrast, for viable seed production, only the effects of total precipitation and mean daily minimum temperature at resource priming were more pronounced with increasing elevation. For both total and viable seed production between 1973 and 2009 (using data from nine elevations), ΔAIC and *P*-values for the interaction of each of the four climate variables with elevation also indicated that the influence of climate change increased with elevation (statistics not given).

**Table 3:** Variability in mean annual total and viable seed production (1966 to 2009) using data from three elevations (1050, 1190, and 1340 m) determined as a function of year, elevation, and climate variables using generalized least squares regression. Climate variables were precipitation (Prec) or temperature (either as mean daily minimum (*T*_min_) or as mean daily maximum (*T*_max_) for resource priming (RP), primordia development (PD), and postflowering (PF)) periods. Reduction in Akaike information criterion (ΔAIC) from a null model and *P*-value(s) are given for variables in each model

	Total seed production		Viable seed production	
Model	ΔAIC	*P*-value(s)	ΔAIC	*P*-value(s)
PrecRP	−5.1	0.008 (PrecRP)	−9.5	<0.001 (PrecRP)
PrecRP + Year	−6.0	0.032 (PrecRP), 0.094 (Year)	−10.7	0.005 (PrecRP), 0.076 (Year)
PrecRP × Elevation	−2.9	0.027 (PrecRP × Elevation)	−4.5	0.011 (PrecRP × Elevation)
*T*_min_RP	−10.0	<0.001 (*T*_min_RP)	−14.2	<0.001 (*T*_min_RP)
*T*_min_RP + Year	−8.9	0.006 (*T*_min_RP), 0.358 (Year)	−13.1	0.001 (*T*_min_RP), 0.357 (Year)
*T*_min_RP × Elevation	−7.8	0.002 (*T*_min_RP × Elevation)	−15.3	<0.001 (*T*_min_RP × Elevation)
*T*_max_PD	−78.5	<0.001 (*T*_max_PD)	−64.8	<0.001 (*T*_max_PD)
*T*_max_PD + Year	−77.5	<0.001 (*T*_max_PD), 0.316(Year)	−63.0	<0.001 (*T*_max_PD), 0.705 (Year)
*T*_max_PD × Elevation	−3.6	0.018 (*T*_max_PD × Elevation)	0.7	0.244 (*T*_max_PD × Elevation)
*T*_max_PF	−48.2	<0.001 (*T*_max_PF)	−30.7	<0.001 (*T*_max_PF)
*T*_max_PF + Year	−46.9	<0.001 (*T*_max_PF), 0.419(Year)	−28.7	<0.001 (*T*_max_PF), 0.822 (Year)
*T*_max_PF × Elevation	−2.9	0.028 (*T*_max_PF × Elevation)	1.1	0.352 (*T*_max_PF × Elevation)

Models which combined the sequence of resource priming total precipitation and floral primordial development mean daily maximum temperature had ΔAICs that were 15.7 and 25.7 units lower for total and viable seed production, respectively, than the best single-variable models using floral primordial development mean daily maximum temperature alone (Table 3; Table 4). There was increased support for the best two variable models when resource priming mean daily minimum temperature was added with ΔAICs that were 12.1 and 16.9 units lower for total and viable seed production, respectively, with *P*-values all <0.001 (Table 4). The model representing the sequence of all four climate variables reduced the AIC for a total seed production model by 3.4 units, but not for a viable seed production model (Table 4).

**Table 4:** Variability in mean annual total and viable seed production (1966 to 2009) using data from three elevations (1050, 1190, and 1340 m) determined as a function of climate variables using generalized least squares regression. Climate variables were precipitation (Prec) or temperature (either as mean daily minimum (*T*_min_) or as mean daily maximum (*T*_max_) for resource priming (RP), primordia development (PD), and postflowering (PF)) periods. Reduction in Akaike information criterion (ΔAIC) from a null model and *P*-value(s) are given for variables in each model

	Total seed production		Viable seed production	
Model	ΔAIC	*P*-value(s)	ΔAIC	*P*-value(s)
PrecRP + *T*_min_RP	−13.3	0.023 (PrecRP), 0.002 (*T*_min_RP)	−21.1	0.003 (PrecRP), <0.001 (*T*_min_RP)
PrecRP + *T*_max_PD	−94.2	<0.001 (PrecRP), <0.001 (*T*_max_PD)	−90.5	<0.001 (PrecRP), <0.001 (*T*_max_PD)
PrecRP + *T*_max_PF	−50.5	0.040 (PrecRP), <0.001 (*T*_max_PF)	−38.4	0.002 (PrecRP), <0.001 (*T*_max_PF)
*T*_max_PD + *T*_max_PF	−90.8	<0.001 (*T*_max_PD), <0.001 (*T*_max_PF)	−68.6	<0.001 (*T*_max_PD), 0.013 (*T*_max_PF)
*T*_min_RP + *T*_max_PD	−92.8	<0.001 (*T*_min_RP), <0.001 (*T*_max_PD)	−84.4	<0.001 (*T*_min_RP), <0.001 (*T*_max_PD)
*T*_min_RP + *T*_max_PF	−48.4	<0.001 (*T*_min_RP), <0.001(*T*_max_PF)	−35.2	0.012 (*T*_min_RP), <0.001 (*T*_max_PF)
PrecRP + *T*_max_PD + *T*_max_PF	−104.1	<0.001 (PrecRP), <0.001 (*T*_max_PD), <0.001 (*T*_max_PF)	−91.8	<0.001 (PrecRP), <0.001 (*T*_max_PD), 0.069 (*T*_max_PF)
PrecRP + *T*_min_RP + *T*_max_PD	−106.3	<0.001 (PrecRP), <0.001 (*T*_min_RP), <0.001 (*T*_max_PD)	−107.4	<0.001 (PrecRP), <0.001 (*T*_min_RP), <0.001 (*T*_max_PD)
PrecRP + *T*_min_RP + *T*_max_PF	−50.1	0.057 (PrecRP), 0.218 (*T*_min_RP), <0.001 (*T*_max_PF)	–41.5	0.005 (PrecRP), 0.026 (*T*_min_RP), <0.001 (*T*_max_PF)
*T*_min_RP + *T*_max_PD + *T*_max_PF	−97.5	0.004 (*T*_min_RP), <0.001 (*T*_max_PD), 0.005 (*T*_max_PF)	−83.2	<0.001 (*T*_min_RP), <0.001 (*T*_max_PD), 0.339 (*T*_max_PF)
PrecRP + *T*_min_RP + *T*_max_PD + *T*_max_PF	−109.7	<0.001 (PrecRP), 0.007 (*T*_min_RP), <0.001 (*T*_max_PD), 0.014 (*T*_max_PF)	−105.6	<0.001 (PrecRP),<0.001 (*T*_min_RP), <0.001 (*T*_max_PD), 0.673 (*T*_max_PF)

## Discussion

We detected a trend of increasing total and viable tree seed production over 45 years, particularly at higher elevations. This long-term trend of increasing seed production was related to a change in four climate variables during three key phases in the reproductive cycle. Greater sensitivity of tree seeding to changes in climate at higher elevations reveals that the response of species to climate change will be spatially variable and contingent on site productivity.

Determining the causes of such temporal trends in tree demographic processes is challenging and can lead to controversial conclusions (e.g., Lewis et al. 2009; Lines et al. 2010). One explanation for a trend in forest productivity is that it reflects compositional, structural, and soil resource availability changes that occur during stand development (e.g., Gower et al. 1996; Clinton et al. 2002). For example, mountain beech population-level seed production can vary among stands that represent various stages of stand development with different soil nutrient availability (Davis et al. 2004; Smaill et al. 2011). Although the forests used in the current study do contain a small-scale (largely <400 m^2^) mosaic of stands at various stages of stand development (Allen et al. 1999; Coomes and Allen 2007; Coomes et al. 2012), the sampling design with widespread seed trays should average across these stages and we do not believe stand development accounts for the structured, elevation-dependent changes reported here. Compositional changes are also not a factor in our study because across our 350-m elevation gradient, there is only one tree species.

Temporal variation in mountain beech total and viable seed production is overall strongly related to climatic patterns. However, the absence of a first-order autoregressive correlation structure in any climate variables, but a presence in seed production, also suggests a level of decoupling of climate from interannual variation in seed production and underscores the importance of resources in determining seed production. Trees take time to recover from seeding events (e.g., Allen and Platt 1990; Crone et al. 2011). Our study confirmed the sequence of total precipitation, and mean daily minimum temperature, during resource priming, mean daily maximum temperature during floral primordia development, and mean daily maximum temperature postflowering all as predictors of total and viable mountain beech seed production (Table 3; Allen and Platt 1990; Schauber et al. 2002; Richardson et al. 2005; Smaill et al. 2011). That climate variables over three consecutive summers relate to seed production supports the importance of a particular temporal sequence of climatic events at a particular location. Reductions in AIC for models containing various combinations of the four significant climate variables were always greatest when the models included resource priming total precipitation as first in the sequence (Table 4). For mountain beech, such climate variables were additive in regression models explaining long-term variation in seed production (Allen and Platt 1990; Richardson et al. 2005). The sequence of climate variables is similar to those related to tree seed production in some Northern Hemisphere forests (e.g., Van Vredenburch and la Bastide 1969; Övergaard et al. 2007; Roland et al. 2014). There was no evidence, as hypothesized, that total mountain beech seed production better related to relatively cool temperatures and high precipitation at the time of resource priming and that viable seed production better related to relatively warm temperatures at the time of floral primordia development or, particularly, postflowering. This contrasts with *Picea glauca*, in the interior of Alaska, where seed viability is strongly and positively related to summer temperatures over the period of primordia development, whereas total seed production is negatively related to summer temperatures over the period of primordia development (Roland et al. 2014).

We believe the 45-year trend in mountain beech seed production is in a major part related to the direct or indirect effect of decadal changes in climate. This is because decadal changes in each of the four climate variables, significantly related to total and viable seed production, were consistent with the temporal trend of increasing seed production (Table 2; Table S1) and that GLS regressions predicting seed production from climate variables subsumed any temporal trends (Table 3). Rising atmospheric CO_2_ partial pressures, of course, covary with the decadal trend in mountain beech seed production. Therefore, it is possible that increasing atmospheric CO_2_ partial pressures are generating an increase in net C availability in mountain beech forest and that this may prime seed production by the trees. Richardson et al. (2005) developed a daily net C availability model for our same mountain beech forest study area based upon net C canopy uptake (Whitehead et al. 2002) and subtracting estimates of wood respiration. Net C canopy uptake was based upon photosynthesis, respiration, and stomatal conductance parameterized for daily estimates of temperature, solar radiation, and precipitation from the Craigieburn Range climate station. The model did not include variability in nutrient availability but did include changes in atmospheric CO_2_ partial pressures. Overall, this model (1973 to 2002) did not support that increasing atmospheric CO_2_ partial pressures caused a trend in net seasonal C availability (Richardson et al. 2005). In addition, our mountain beech forest occurs on cool, moist sites with low-nutrient availability and such sites are unlikely to exhibit an atmospheric CO_2_ partial pressure fertilization effect (e.g., Körner 2006; Huang et al. 2007; Millard et al. 2007; Palacio et al. 2014). In fact, because nutrient limitation increases with elevation, our study shows decadal increases in seed production on those sites least likely to display a CO_2_ partial pressure fertilization effect.

A striking result is the greater temporal increase of mountain beech seed production with elevation and the greater influence of the four significant climate variables with increasing elevation (Fig. 3; Table 3, Table S1). This was expected for resource priming total precipitation and postflowering maximum daily temperature as they have previously been shown to more strongly influence seeding at higher elevations (Allen and Platt 1990; Richardson et al. 2005). Greater phenological sensitivity of plants to climate variation at higher elevations has also been shown in Scottish mountains (Chapman 2013). For mountain beech, Richardson et al. (2005) modeled that summer net C availability is greater in years when soils are moist (greater total precipitation) and mean daily minimum temperature is low. These authors also showed that net C availability at resource priming had a stronger, positive relationship to total seed production near tree line than at lower elevations (Richardson et al. 2005). This, in combination with our study showing increased summer precipitation and decreased summer mean daily minimum temperature at the time of resource priming (that lead to increased net C availability), suggests net C availability as one possible direct mechanism behind the greater increase in seed production at higher elevations. However, it has been shown that seeding by tree species is independent from old carbon reserves and instead trees use current assimilates (e.g., Hoch et al. 2013). It may be that high net C availability at resource priming is instead allocated by trees to stimulate mycorrhizal activity and thus increase nutrient availability (e.g., Smith and Read 2008; Högberg et al. 2010).

Another potential indirect mechanism behind our increased seed production at higher elevations lies in the interplay between climate and nutrient availability. Resource priming total precipitation was always first in the best-supported sequence of climate variables predicting seed production (Table 4), and greater summer precipitation can lead to more litter mass loss (e.g., Upadhyay et al. 1989) and increased N mineralization and uptake (e.g., Paul et al. 2003; Smaill et al. 2011; Schaeffer et al. 2013). Although nutrient availability can decline with elevation, because of decreased organic matter decomposition and nutrient mineralization (e.g., Sundqvist et al. 2013), it has also been shown that nitrogen availability can increase with elevation in summers with high soil moisture status (Groffman et al. 2009) potentially reflecting the ectomycorrhizal stimulation discussed above. We suggest that nitrogen availability could also be more responsive to changes in summer precipitation at higher elevations in mountain beech forest. Smaill et al. (2011) have shown that greater resource priming precipitation in our same mountain beech forest study area elevated nitrogen uptake in the dry summer months. In contrast, experimental addition of nitrogen to mountain beech forest soils increased seed production in most years but markedly reduced the importance of resource priming precipitation as a proximate factor (Davis et al. 2004; Smaill et al. 2011). Enhanced nitrogen uptake with greater resource priming precipitation potentially leads to increased internal storage which is subsequently remobilized for primordial development in the following growing season, particularly in relatively warm summers. The ability to store and mobilize internal nitrogen resources is a fundamental aspect of nutrient dynamics in perennial plants (Millard and Grelet 2010). Nutrients can increase seed production directly by allowing more nitrogen to be allocated to reproductive tissue development (e.g., Chandler 1938; Davis et al. 2004; Han et al. 2008, 2014) or by enhancing photosynthesis and the supply of fixed carbohydrates for growth (e.g., Waring and Schlesinger 1985). The demands of seeding events can subsequently deplete stored nutrients in trees (e.g., Sala et al. 2012; Ichie and Nakagawa 2013) and may explain the autoregressive correlation of seeding in our study. More generally, nutritional status has an important mechanistic control over plant flowering and seed production (e.g., Danielson and Frommer 2013).

Our finding of spatially variable seed production responses to decadal changes in climate adds to an emerging view that differences in the proximate factors controlling flowering and seed production across the landscape are important elements in understanding seed production patterns (e.g., Crone et al. 2011; Cook et al. 2012; Koenig and Knops 2014; Roland et al. 2014). As Koenig and Knops (2014) argue, for acorn production by oaks, our results support a view that there is no single, unified environmental driver of seed production within taxa. This may explain why, at best, only modest relationships have sometimes been found between specific climate variables and temporal variation in seed production (e.g., Schauber et al. 2002; Fearer et al. 2008; Kelly et al. 2013; Koenig and Knops 2014). Koenig and Knops (2014) suggest one option is that the diversity of environmental factors related to seeding could in fact be arbitrary and unrelated to the physiology of seed production – hence the factors at a location act merely as a cue. We support a view that differences in the proximate factors controlling seed production across the landscape are sometimes physiologically significant and that soil nutrient availability is one such resource likely to explain landscape patterns in seed production (e.g., Davis et al. 2004; Smaill et al. 2011; Tanentzap et al. 2012; Canham et al. 2014).

Because variation in tree seed production has significant ecosystem-level consequences, it is crucial to develop an understanding of this variability that is highly predictive and as far as possible mechanistic. This understanding needs to be highly predictive because at times, large financial resources are allocated for, as examples, responding to human health and biodiversity threats resulting from seeding events (e.g., Jones et al. 1998). Our results support a view that capturing the responsive elements of seed production to a changing climate and developing predictive landscape-level models will require simultaneously accommodating a diversity of environmental variables (e.g., Cook et al. 2012; Koenig and Knops 2014; cf. Kelly et al. 2013), that drivers are modified by resource availability (e.g., Tanentzap et al. 2012), and that multiple drivers are important sequentially during reproductive phases (Eis 1973; Allen and Platt 1990; Piovesan and Adams 2005). Developing a mechanistic basis will improve our ability to manage ecosystem-level consequences. For example, if nutrient availability is a critical driver at certain stages in the reproductive cycle, we might choose to promote seeding through fertilizer addition when it benefits breeding success of threatened birds. Similarly, we might inhibit seeding through nutrient immobilization where seeding events have negative impacts for human health or biodiversity. Such options may well be fruitful under a changing climate.

## Supporting Information

Table S1 Estimated model coefficients (±SE in parentheses) for GLS models fitted to mean annual total and viable seed production data over the 1965–2009 period.
