# Supplementary material for: Elevation-dependent responses of tree mast seeding to climate change over 45 years
Source: Ecol Evol. 2014 Aug 28;4(18):3525–37. doi: 10.1002/ece3.1210 (PMC4224528; doi:10.1002/ece3.1210)
Supplement: Table S1 — Estimated model coefficients (±SE in parentheses) for GLS models fitted to mean annual total and viable seed production data over the 1965–2009 period. [file ece30004-3525-sd1.docx]

Appendix 1

Table S1. Estimated model coefficients (±SE in parentheses) for GLS models fitted to mean annual total and viable seed production data over the 1965–2009 period. Total and viable seedfall data were transformed using log_10_(seedfall +1).

**Model Intercept (±SE) Year (±SE) Climate variable (±SE) Climate×elevation interaction (±SE)**

Total seed production

Null 2.35 (0.0713)

PrecRP 1.698 (0.254) 0.00155 (0.000578)

PrecRP + Year –17.6 (11.4) 0.00977 (0.00579) 0.00128 (0.000591)

PrecRP × Elevation 1.84 (0.243) 0.00000103 (0.00000046)

TminRP 4.798 (0.696) –0.382 (0.108)

TminRP + year –6.65 (12.4) 0.00561 (0.00607) –0.333 (0.1203)

TminRP × Elevation 3.91 (0.495) –0.000203 (0.0000643)

TmaxPD –10.8 (1.24) 0.716 (0.0675)

TmaxPD + Year –1.38 (9.51) –0.00502 (0.00499) 0.747 (0.0725)

TmaxPD × Elevation 0.754 (0.669) 0.000073 (0.0000303)

TmaxPF –5.58 (0.948) 0.405 (0.0483)

TmaxPF + Year 1.45 (8.73) –0.00373 (0.0046) 0.424 (0.0537)

TmaxPF × Elevation 1.001 (0.614) 0.0000579 (0.0000261)

Viable seed production

Null 1.59 (0.0725)

PrecRP 0.731 (0.259) 0.00203 (0.000591)

PrecRP + Year –19.6 (11.4) 0.0103 (0.00574) 0.00173 (0.000606)

PrecRP × Elevation 0.973 (0.2502) 0.00000122 (0.000000476)

TminRP 4.54 (0.716) –0.461 (0.111)

TminRP + Year –7.03 (12.5) 0.00565 (0.00611) –0.408 (0.125)

TminRP × Elevation 3.68 (0.491) –0.000274 (0.0000637)

TmaxPD –11.3 (1.38) 0.699 (0.07503)

TmaxPD + Year –7.61 (9.81) –0.00196 (0.00517) 0.713 (0.0813)

TmaxPD × Elevation 0.793 (0.685) 0.0000364 (0.0000311)

TmaxPF –5.38 (1.11) 0.355 (0.0567)

TmaxPF + Year –7.69 (10.3) 0.00123 (0.00543) 0.349 (0.0636)

TmaxPF × Elevation 0.991 (0.645) 0.0000256 (0.0000274)
